# Spontaneous Aortic Dissection Limited to the Sinus of Valsalva: Report of Two Cases 

**Published:** 2016-01-13

**Authors:** Maryam Nabati, Babak Bagheri, Samira Eslami, Razhan Piran

**Affiliations:** 1 *Department of Cardiology, Faculty of Medicine, Mazandaran University of Medical Sciences, Sari, Iran.*; 2 *Student Research Committee, Faculty of Medicine, Mazandaran University of Medical Sciences, Sari, Iran.*

**Keywords:** *Aorta*, *Dissection*, *Aortic valve insufficiency*, *Sinus of valsalva*, *Echocardiography, transesophageal*

## Abstract

Dissection of the sinus of Valsalva is a rare and life-threatening event. It often occurs during percutaneous coronary intervention in the right coronary artery (RCA). Dissection flap usually involves the RCA and the right sinus of Valsalva. Here we report two extremely rare cases of spontaneous dissection limited to the non-coronary sinus of Valsalva, causing severe aortic valve regurgitation: a male aged 51 years presenting with back pain, weakness, and presyncope and another male aged 71 years presenting with orthopnea and weakness. The dissection was found by transesophageal echocardiography. Surgical treatment was successful for both patients.

One year after surgery, both patients were asymptomatic and follow- up transthoracic echocardiography did not show any abnormality.

## Introduction

Aortic dissection is a serious cardiovascular accident. The ascending aorta is adjacent to the heart, so its involvement is more dangerous.^1^ The dissection of the sinus of Valsalva is a rare and life-threatening event and it may occur during percutaneous coronary intervention.^2^

We report two cases of spontaneous dissection of the non-coronary sinus of Valsalva, leading to severe aortic regurgitation diagnosed by transesophageal echocardiography (TEE). Neither of the cases involved the coronary arteries. To the best of our knowledge, the current literature lacks cases with non-coronary sinus involvement diagnosed via preoperative TEE.

## Case Report

***Case # 1***

A 51-year-old man presented with back pain, severe weakness, and presyncope attacks one month prior to admission. The patient was a smoker (25 packs/year). He had hypertension and previously took 100 mg of Atenolol daily. At admission, his blood pressure was 170/95 mmHg, and his heart rate was 65 beats per minute. A high-frequency early diastolic blowing murmur was heard at the left sternal border and apex. No other abnormalities were found on physical examination.

The electrocardiogram (ECG) obtained at admission showed non-specific T-wave inversion in leads I, II, aVL, V5, and V6.

**Figure 1:**
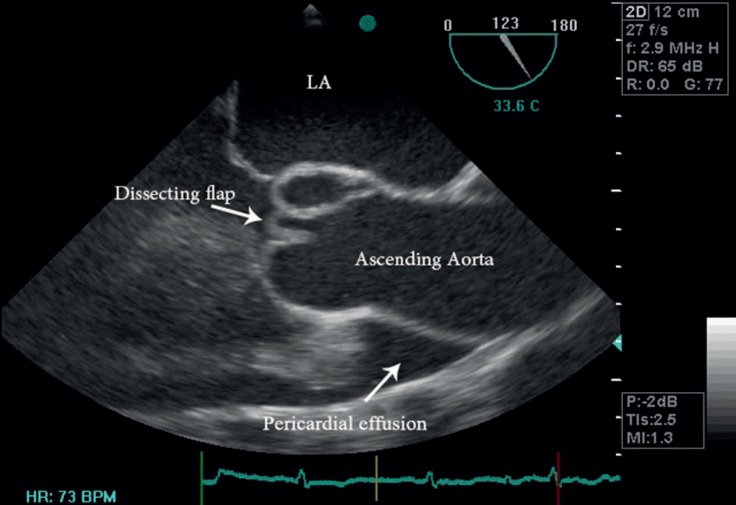
Transesophageal echocardiography in the mid-esophageal view, demonstrating a linear dissection flap originating from the non-coronary sinus of Valsalva

TEE showed a significant amount of pericardial effusion and severe aortic regurgitation (AR). Also, there was early diastolic collapse of the right ventricular outflow tract, consistent with tamponade physiology (Figure 1). Left ventricular systolic function was preserved, and left ventricular diastolic volume was severely increased. Systolic pulmonary artery pressure was approximately 50 mmHg, and the ascending aorta was mildly dilated (3.75 cm). Moreover, aortic valve annulus diameter was 2.36 cm, Valsalva sinus diameter was 3.94 cm, and sinotubular junction diameter was 2.96 cm (Figure 1). TEE demonstrated a linear dissection flap in the non-coronary sinus of Valsalva (Figure 1) with protrusion into the aortic valve, leading to the maladaptation of the aortic valve and subsequent severe AR. 

Coronary angiography was performed safely and showed normal coronary arteries without any significant stenosis or rupture. The patient underwent open heart surgery. The surgeon confirmed a localized dissection flap in the non-coronary sinus of Valsalva. The patient successfully underwent the Bentall procedure and was asymptomatic during 6 months of follow-up.

***Case # 2***

A 71-year-old man complained of orthopnea and weakness two months prior to admission. He had a 5-year history of hypertension and no history of cardiovascular disease. The patient was a non-smoker and had no history of diabetes mellitus or dyslipidemia. At admission, his blood pressure was 150/90 mmHg, and his heart rate was 63 beats per minute. A diastolic blowing murmur was heard at the left sternal border. 

**Figure 2:**
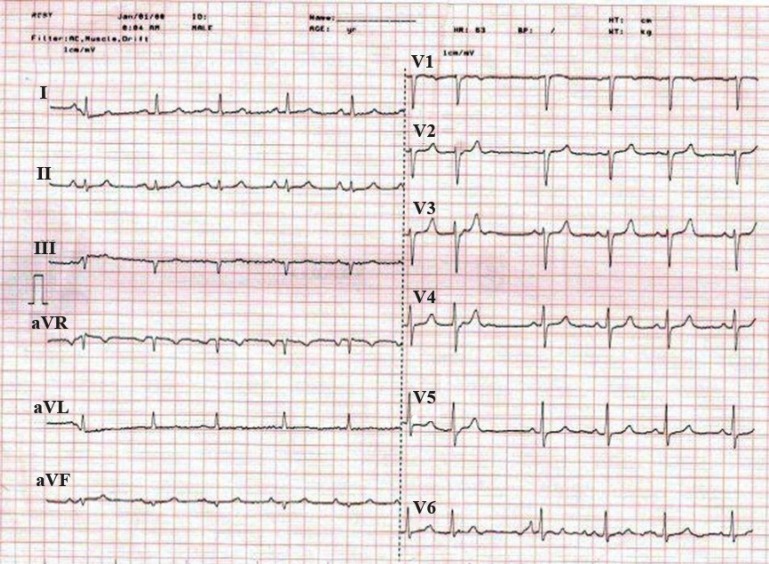
Electrocardiogram at admission, showing sinus rhythm, normal axis, QS-waves in leads II and aVF, and inverted T wave in aVF

His ECG showed QS-waves in leads III and aVF and inverted T wave in aVF, but no another abnormality (Figure 2). 

**Figure 3:**
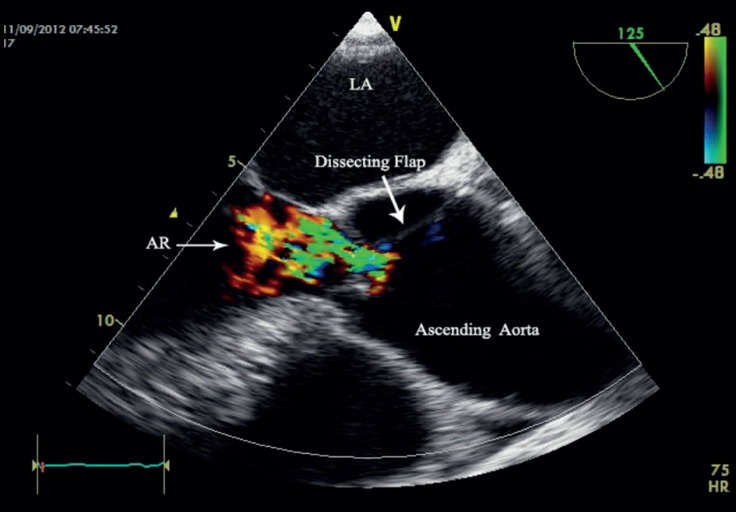
Transesophageal echocardiography in the mid-esophageal view, showing severe aortic regurgitation (AR) and a dilated ascending aorta

**Figure 4:**
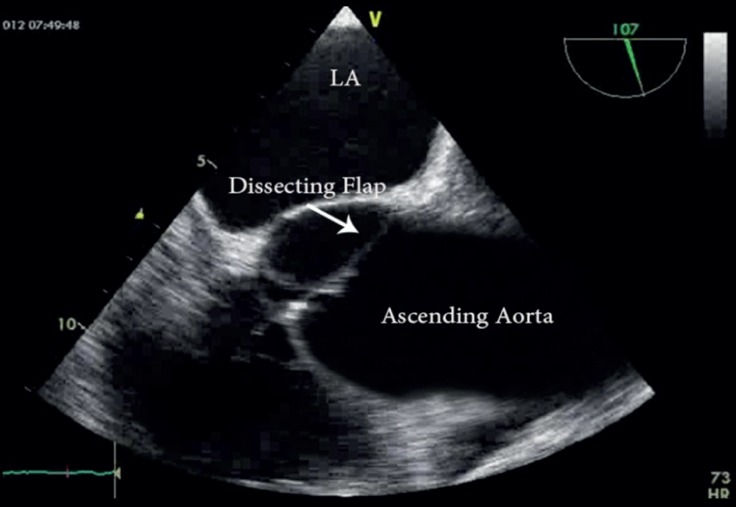
Transesophageal echocardiography in the mid-esophageal view, showing a linear dissection flap in the non-coronary sinus of Valsalva

TEE showed severe AR and a dilated ascending aorta (5 cm) (Figure 3). Also, the aortic valve annulus diameter was 2.4 cm, Valsalva sinus diameter was 4 cm, and sinotubular junction diameter was 4 cm. Left ventricular systolic function was preserved. Left ventricular diastolic volume was mildly increased. Similar to the previous case, TEE showed a linear dissection flap in the non-coronary sinus of Valsalva with protrusion into the aortic valve, leading to severe AR (Figure 4).

Coronary angiography was performed safely and showed normal coronary arteries. Cardiopulmonary bypass was instituted through a midline sternotomy. The aortic valve was repaired, and the ascending aorta was replaced using a Dacron graft. Postoperative echocardiography showed no residual AR, and the clinical course was uneventful.

LA, Left atrium

LA, Left atrium

## Discussion

Localized dissection of the sinus of Valsalva is a rare event which may occur during catheterization interventions. It may cause dangerous complications such as the dissection of the ascending aorta.^3^

In 1999, Ishibashi et al.^4^ reported a case of non-coronary sinus of Valsalva dissection diagnosed only during surgery. The patient was admitted with chief complaints of cough and thoracic distress. In 2006, Vianna et al.^1^ reported a rare variation of type-A dissection. The dissection was limited to the left sinus of Valsalva and caused severe obstruction of the left main coronary artery. A flap in the left sinus of Valsalva was determined by TEE and high-slice system computed tomography scan. This patient was admitted with sudden onset of chest pain and profuse sweating. There was no associated aortic valve involvement and AR.

Our first patient was admitted with back pain, severe weakness, and presyncope. In the second patient, the presenting symptoms were orthopnea and weakness. Weakness and orthopnea seem to be due to the involvement of the aortic valve and severe AR, leading to heart failure. Also, the presyncope attacks in the first patient were probably due to cardiac tamponade.

Chronic hypertension causes intimal thickening, necrosis of smooth muscle cells, and fibrosis of elastic structures of the vessel wall, leading to stiffness and vulnerability to pulsatile flow and inducing a substrate for aneurysm formation and dissection. Also, smoking, dyslipidemia, and cocaine use are other risk factors. Furthermore, inflammatory diseases can destroy the medial layers of the aorta and lead to aneurysm formation and dissection of the aortic wall. Inherited connective tissue disorders such as Marfan's syndrome are other known risk factors.^5^

In our patients, autoimmune and inflammatory diseases were not present, and nor was there any evidence of inherited connective tissue disorders. Among common cardiovascular risk factors, both patients were male and had poorly controlled hypertension. Also, the first patient was a smoker.

In our pathologic examination of the dissection area, the aorta was normal without significant protruding or non-protruding atherosclerotic plaques. However, the aortic wall was severely thin (impending to rupture) in the involved area. Localized dissection was produced spontaneously without involving the coronary arteries, prior to coronary angiography. Also, the dissecting flaps protruded into the aortic valve, leading to acute severe AR.

To the best of our knowledge, these forms of spontaneous localized dissection of non-coronary sinus of Valsalva, diagnosed before surgery by TEE, have not been reported previously.

## Conclusion

Localized dissection of the Valsalva sinus should be considered in the differential diagnosis of acute severe aortic regurgitation.

## Notes:

***This paper should be cited as:*** Nabati M, Bagheri B, Eslami S, Piran R. Spontaneous Aortic Dissection Limited to the Sinus of Valsalva: Report of Two Cases. J Teh Univ Heart Ctr 2016;11(1):34-37.
